# Convergent and parallel evolution in life habit of the scallops (Bivalvia: Pectinidae)

**DOI:** 10.1186/1471-2148-11-164

**Published:** 2011-06-14

**Authors:** Alvin Alejandrino, Louise Puslednik, Jeanne M Serb

**Affiliations:** 1 Department of Ecology, Evolution, and Organismal Biology, Iowa State University, Ames, Iowa, 50011, USA; 2 Institute for Conservation Biology and Environmental Management, School of Biological Sciences, University of Wollongong, New South Wales, 2519 Australia

## Abstract

**Background:**

We employed a phylogenetic framework to identify patterns of life habit evolution in the marine bivalve family Pectinidae. Specifically, we examined the number of independent origins of each life habit and distinguished between convergent and parallel trajectories of life habit evolution using ancestral state estimation. We also investigated whether ancestral character states influence the frequency or type of evolutionary trajectories.

**Results:**

We determined that temporary attachment to substrata by byssal threads is the most likely ancestral condition for the Pectinidae, with subsequent transitions to the five remaining habit types. Nearly all transitions between life habit classes were repeated in our phylogeny and the majority of these transitions were the result of parallel evolution from byssate ancestors. Convergent evolution also occurred within the Pectinidae and produced two additional gliding clades and two recessing lineages. Furthermore, our analysis indicates that byssal attaching gave rise to significantly more of the transitions than any other life habit and that the cementing and nestling classes are only represented as evolutionary outcomes in our phylogeny, never as progenitor states.

**Conclusions:**

Collectively, our results illustrate that both convergence and parallelism generated repeated life habit states in the scallops. Bias in the types of habit transitions observed may indicate constraints due to physical or ontogenetic limitations of particular phenotypes.

## Background

When two species occupy comparable trophic niches, similar phenotypes can be generated via analogous evolutionary responses [1-4]. As a consequence, repeated phenotypes have long been treated as evidence for adaptation at the macroevolutionary scale [5-9]. Two important patterns in iterative morphological evolution are convergence and parallelism, which can be distinguished by examining the phenotypic trajectories along a phylogeny [10]. Evolutionary convergence is implicated when two or more lineages with different ancestral phenotypes independently evolve along different trajectories towards the same adaptive phenotype; whereas, evolutionary parallelism is revealed when independent lineages with comparable ancestral morphologies evolve towards a new, but similar, phenotype. Importantly, the application of a phylogenetic approach to discern between convergence and parallelism alleviates some of the operational difficulties of separating these two concepts, thereby allowing a meaningful, quantitative way of assessing repeated evolutionary patterns (for reviews of this highly contested issue see: [11-13]).

The best known studies examining repetitive evolutionary patterns include morphological, ecological, and behavioral traits in all major vertebrate lineages (e.g., fishes: [3,14]; amphibians: [15]; reptiles: [16,17]; birds: [18,19]; mammals: [20]). To a lesser extent, similar work has been done in invertebrate groups, specifically arthropods. For example, convergent or parallel evolution has been identified in replicated shifts of host use in insects [21,22], web construction in arachnids [8], larval morphology and antipredator behavior in aquatic insects [23], and adult morphology in barnacles [24]. Outside of arthropods, few studies using invertebrates explicitly test for convergence and parallelism (but see gastropods: [25,26]; bivalves: [27]). Indeed, if the patterns seen in vertebrates are representative, it suggests that repetitive patterns of phenotypic evolution should be far more prevalent across the animal kingdom than is currently recognized, as vertebrates comprise only 5 percent of all animal diversity.

Here we use scallops as a non-arthropod invertebrate model to study convergence and parallelism. Scallop species comprise a large family (Pectinidae) of 264 recognized species and are found globally in a wide range of marine habitats from the intertidal zone to depths of 7000 meters (m) [28,29]. Scallops exhibit a diverse set of life habits that are related to the animal's ecological requirements and behavioral attributes [30] and are organized into six categories based on the methods and permanence of attachment to a substrate, locomotive ability, and spatial relationship to a substrate (epifaunal versus semi-infaunal; see Table 1). Species are categorized by the life habit displayed during adulthood and membership to a life habit class typically precludes the display of other habits. Recent work by Smith and Jackson [31] has demonstrated the evolutionary importance of pectinid life habit by linking environmental factors to the diversification or decline of lineages.

**Table 1:** Descriptions of life habit classes in the Pectinidae

Life habit	Description	Genera included in study	References
Nestle	Settle and byssally attach to living *Porites *corals; coral grows around and permanently contains scallop	*Pedum*	[38,61]

Cement	Permanently attaches to hard or heavy substratum as new shell is generated	*Crassadoma*, Talochlamys*	[62]

Byssal attach	Temporarily attaches to a substratum by byssus threads; can release and reorient	*Azumapecten*, Brachtechlamys*, *Caribachlamys*, *Chlamys*, *Coralichlamys*, *Cyclopecten*, *Excellichlamys*, *Gloripallium*, *Laevichlamys*, *Leptopecten*, Mimachlamys*, *Pascahinnites*, *Scaeochlamys*, *Semipallium*, *Spathochlamys*, Talochlamys*, *Veprichlamys*, *Zygochlamys*	[39]

Recess	Excavates cavity in soft sediment; full/partial concealment	*Euvola*, *Mizuhopecten*, *Pecten*, *Patinopecten*	[39,40]

Free-living	Rests above soft sediment or hard substratum	*Aequipecten*, *Anguipecten*, *Annachlamys*, *Argopecten*, Brachtechlamys*, *Cryptopecten*, *Decatopecten*, *Delectopecten*, *Equichlamys*, Mimachlamys*, *Mirapecten*, *Nodipecten, Pseudamussium*	[39]

Gliding	Able to swim > 5 m/effort; includes a level swimming phase with a glide component	*Adamussium*, *Amusium*, "*Amusium*," *Placopecten*	[44-46]

Species-specific classes are the predominant life habit exhibited by sexually mature individuals listed from least to most active. An asterisk indicates multiple life habits are exhibited within the genus.

In this paper, we employ a phylogenetic framework to examine the evolution of species-specific life habit categories in the scallops. We have generated the most comprehensive multigene phylogeny of the Pectinidae to date in order to determine the number of independent origins of each life habit class. We then distinguished between convergent and parallel trajectories of life mode evolution by applying a phylogenetically-based approach [10] to answer the following questions: How repetitive is the evolution of life mode in the scallops? When a life habit has multiple origins, are these lineages the result of convergent evolution or parallel evolution? Are particular transitions between life habit classes more likely than others? Our results demonstrate that five of the six life habit types exhibited by scallops have evolved multiple times. We identified 17 repeated transitions between life habit classes within the Pectinidae that were the result of both parallel and convergent evolution. Interestingly, despite repeated evolutionary transitions, we found that not all life habit classes function as progenitor states in the scallops.

## Methods

### Phylogenetic analysis

We examined 81 species, representing 31% of extant taxa from the Pectinidae. Taxonomic classification follows that of Dijkstra [28] and Waller [29]. Eleven species from three closely allied families, Propeamussiidae, Limidae, and Spondylidae, were included as outgroup taxa based on the results from [32]. All specimens were preserved in 95% ethanol and were provided by either museum collections or colleagues. When possible, DNA was extracted from two or more individuals per species as a test for congruent placement in the phylogenetic analyses.

Previously, nuclear Histone H3 and mitochondrial 12S rRNA and 16S rRNA gene fragments were amplified for 39 taxa by Puslednik and Serb [32]. Here, we build on their three-gene dataset by adding 53 more species and a nuclear gene region, 28S rRNA. Primer sequences for 12S rRNA, 16S rRNA, and Histone H3 and PCR and sequencing conditions are described in Puslednik and Serb [32]. We designed new primers for the 28S rRNA region for this study (sc28S_70F: 5'-CAGCACCGAATCCCTCAGCCTTG-3', sc28S_950R: 5'-TCTGGCTTCGTCCTACTCAAGCATAG-3', 28S_Limoida_121F: 5'-TCAGACGAGATTACCCGCTGAATTTAAGC-3'). When the PCR optimization steps failed to amplify a significant amount of product (< 20ng/μl) or a single product, we cloned the PCR products following manufactures instructions (TOPO Cloning Kit, Invitrogen). Sequencing was carried out in an ABI 3730 Capillary Electrophoresis Genetic Analyzer at the Iowa State University DNA Sequencing Facility. All sequences are deposited in Genbank (accession numbers: HM485575-HM485578, HM535651-HM535659, HM540080-HM540106, HM561991-HM562003, HM600733-HM600765, HM622672-HM622722, HM630371-HM630556; see also Additional file 1, Table S1). Sequences were aligned using CLUSTAL W [33] with a gap-opening penalty of 10.00 and a gap-extending penalty of 0.20 in Geneious Pro [34]. Due to ambiguous alignment, a 169 base pair (bp) hypervariable region in the 16S rRNA gene fragment was excluded from phylogenetic analyses.

Aligned sequences (2438 bp) were partitioned according to locus and codon position for the protein-coding gene Histone H3. For each partition, an appropriate nucleotide substitution model was selected on the basis of the hierchical Likelihood Ratio Test (hLRT) and the Akaike Information Criterion (AIC) using ModelTest 3.7 [35]. Both tests agreed on the GTR+G model for the 12S rRNA partition and the GTR+G + I model for 16S rRNA, 28S rRNA, and Histone H3 partitions. All partitions were analyzed simultaneously as a mixed model Bayesian analysis in MrBayes 3.1.2 [36]. We used the Metropolis Coupled Markov Chain Monte Carlo method with one cold and three hot chains for 5 million generations, sampling every 100th generation for three simulations. The number of generations required to attain stationarity was estimated when the standard deviation of split frequencies fell below 0.01. All trees prior to stationarity were discarded as burn-in and the remaining trees were used to compute a majority-rule consensus topology, branch lengths, and posterior probabilities (PP). Maximum Likelihood (ML) was executed in PhyML 3.0 [37] using the GTR+G + I model. The ML analyses consisted of 1000 replicates and clade support was assessed with 100 bootstrap (BP) pseudoreplicates.

### Life habit classes

Scallops exhibit a diversity of species-specific life habits that range from permanent attachment to or within a substrate to mobile species able to swim continuously over long distances in a single effort. We divided behaviors exhibited by sexually mature individuals into six categories. Byssal-attachers retain the ability to produce a temporary protein fastening, the byssus, into adulthood. Nestling species also attach with a byssus, but differ in that the scallop eventually becomes permanently confined within a cavity of living corals or sponges [38]. Other scallop species are cementers that permanently fasten onto hard substrates through the secretion of new shell material. In contrast, free-living pectinids rarely attach as adults and many species are unable to secrete a byssus once the shell takes on the adult morphology. Whereas free-living species passively occupy a position on or partially covered in soft or sandy substrates, recessers actively construct a saucer-shaped depression in the substrate in which the animal resides so that the upper (left) valve is level or just below the sediment surface [39,40]. The most mobile life habit class is gliding. Although all non-permanently attached species have the ability to swim for short distances (< 1 m) to escape predators [39] or to move between desirable habitats [41], few species can swim greater than 5 m in a single swimming effort before the animal sinks passively to the substrate [39]. Gliding (5 - 30 m/effort) is distinguished from a common swimming response by the presence of a level swimming phase, where the animal is able to maintain a near horizontal trajectory above the substrate [42-44]. The level swimming phase also contains a glide component, where the animal is propelled forward while the valves are held closed [44-46]. Neither a level swimming phase nor a glide component is present in short distance swimming [44,47,48], making gliding a unique life habit state among scallop species.

### Analysis of life habit evolution

Life habit data for extant species of Pectinidae and outgroup taxa were assembled via a review of the literature and supplemented with the personal observations of collectors. Species from outgroup families Propeamussiidae and Limidae are treated as byssal attachers: Waller [49] speculated that the typical habit of the Propeamussidae is to actively secrete a byssus based on the presence of a byssal notch in the adult, while species of the Limidae have been directly observed to byssally attach or build nests made of byssus threads [50,51]. In scallops, classifying life habit involves distinguishing between active versus passive actions of an adult organism. So while most species are able to attach with a byssus for a period of time as juveniles or swim short distances as an escape response, these activities do not determine the life habit of the adult animal. Thus, species were placed into life habit classes based on active and prominent responses of the adult animal to its environment. For example, some species are primarily epifaunal, but are passively buried in soft substrates due to the accumulation of sediment. However, since these species do not actively bury, they are treated as free-living and not recessing species. Life habit assignment for each species is given in Additional file 2, Table S2. Life habits were organized into six states and a character matrix was constructed using standard categorical data (0, unknown behavior; 1, cementing; 2, byssal attaching; 3, free-living; 4, recessing; 5, gliding; and 6, nestling). Brief definitions of life habits are provided in Table 1.

We then reconstructed ancestral states on the Bayesian topology using parsimony and likelihood reconstruction methods in Mesquite 2.6 [52]. Changes between states were unordered. The one parameter Markov k-state (Mk1) model was applied in the likelihood analysis and assumes a single rate for all character state transitions [53]. Likelihood-ratio tests of respective nodes determined the best estimate of the state. Differences in log-likelihood values greater than 2.0 were used to reject the higher negative log-likelihood value, while values less than 2.0 were treated as ambiguous character-state reconstructions.

Finally, to test the null hypothesis that transitions between life habit states (permanent attachment, byssal attaching, free-living, recessing, and gliding) are equally likely to come from any of the five states, we used a Chi-square test to compare the number of the observed to the expected transitions. The test follows an asymptotic chi-squared distribution with four degrees of freedom.

## Results

**Figure 1:**
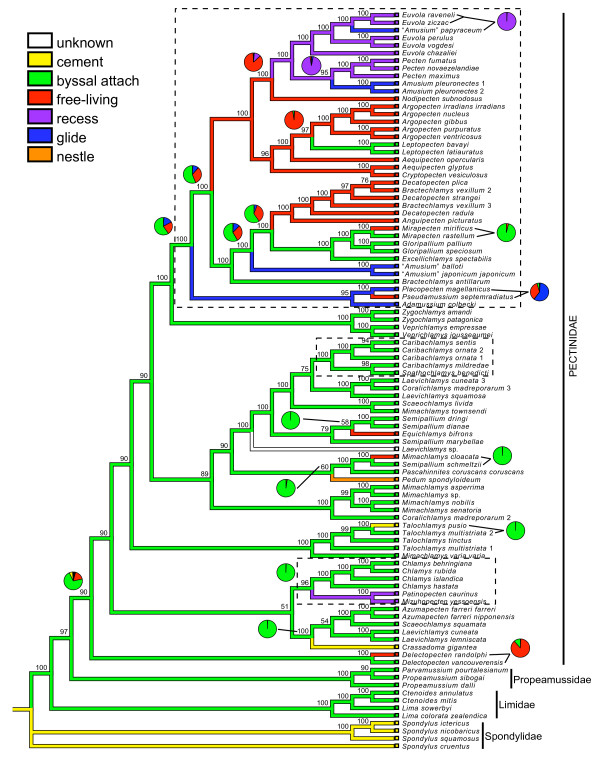
**Bayesian Inference majority-rule consensus topology**. Posterior probability support values (> 50) above respective nodes. Branch colors represent MP reconstruction of life habit and pie charts represent their relative probabilities from ML reconstructions. If probability of ML reconstruction equals 1.0, no pie chart is given. ML ancestral state reconstructions are used to illustrate the 17 life habit transitions described in the text. Dashed boxed represent densest taxonomic sampling.

Of the six life habits examined, byssal attaching is the most common state and is represented by 42 species (52%). Of the remaining species, 21 (25%) are free-living, 10 (12%) species recess, and eight (9%) species glide. In our sample, we included one of the two extant species that exhibit nestling (*Pedum spondyloideum*) and two of the five extant species that cement to a substrate (*Crassadoma gigantea *and *Talochlamys pusio *[= *Chlamys distorta*]). These proportions of non-byssate life habit categories in our taxonomic sample are similar to their representation across the family (free-living = 16.3%; recessing = 12.1%; gliding = 3%; nestling = 0.75%; cementing = 1.9%), where 66% of all species byssally attach (data not shown). Phylogenetic relationships among these species were congruent in both BI (Figure 1) and ML (Additional file 3, Figure S1) topologies except for the placement of three lineages: the *Scaeochlamys livida + Mimachlamys townsendi *clade, the basal clade of the non-*Delectopecten *scallops, and the *Nodipecten subnodosus *lineage. Of these, the only the placement of *N. subnodosus *alters the ancestral state estimation (see below).

To investigate the number of independent origins of life habit categories, we reconstructed ancestral states assuming a Markov model of character evolution with a single parameter to describe the rate of change on the BI topology (Figure 1). For the species analyzed here, ML estimations of ancestral states (pie charts in Figure 1) identify a minimum of 17 transitions between life habit classes (Table 2). These transitions include two origins of recessing, seven origins of the free-living condition, four separate lineages of gliding, and three occurrences of permanent (non-byssal) attachment through either cementation or enclosure within living corals. Byssal attachment was the most likely ancestral state of the Pectinidae and originates a second time in the phylogeny from a free-living ancestor in the *Leptopecten *lineage (Figure 1). Gliding occurs in three genera: *Amusium *(4 species in the genus), *Adamussium *(a monotypic genus) and *Placopecten *(a monotypic genus). Our analysis included three of the four currently recognized species in *Amusium *(= "*Amusium*") genus. Because *Amusium *did not form a monophyletic clade in either BI or ML topologies, these species represent three separate origins of gliding (Figure 1; Additional file 3, Figure S1). The fourth origin of gliding includes the monotypic genera *Adamussium *and *Placopecten*.

**Table 2:** Transitions between life habit states determined from ancestral state reconstruction on the Bayesian topology

Behavioral transition	Number of observed
Recessing to permanent attachment	0

Recessing to byssal attachment	0

Recessing to free-living	0

Recessing to gliding	2

	

Permanent attachment* to byssal attachment	0

Permanent attachment to free-living	0

Permanent attachment to recessing	0

Permanent attachment to gliding	0

	

Byssal attachment to permanent attachment	3 (2 cementing; 1 nestling)

Byssal attachment to free-living	6

Byssal attachment to recessing	1

Byssal attachment to gliding	2

	

Free-living to permanent attachment	0

Free-living to byssal attachment	1

Free-living to recessing	1

Free-living to gliding	0

	

Gliding to permanent attachment	0

Gliding to byssal attachment	0

Gliding to recessing	0

Gliding to free-living	1

**Total number of transitions**	**17**

*Cementing and nestling are grouped together under permanent attachment.

We then examined the number of convergent versus parallel evolutionary events that lead to a particular life habit using phylogenetically-based definitions of convergence and parallelism [10]. Of the 17 life habit transitions, the majority (12; 70%) originated from byssate ancestors and were cases of parallel evolution. Nearly all transitions are repeated at least twice in the phylogeny (Table 2; Figure 1). Six of the seven origins of the free-living state were parallel trajectories arising from byssal attaching ancestors. Likewise, the cementing life habit in *Crassadoma gigantea *and *Talochlamys pusio *lineages arose in parallel from byssal attaching ancestors. The gliding life habit arose in four independent lineages along both parallel and convergent trajectories. "*Amusium*" *papyraceum *and *Amusium pleuronectes *arose in parallel from recessing ancestors, while the "*A*." *balloti *+ "*A*." *japonicum *clade and *Adamussium + *(*Pseudamussium *+ *Placopecten*) clade arose in parallel from byssal attaching ancestors. The recessing life habits of the *Euvola *+ *Pecten *clade and the *Patinopecten *+ *Mizuhopecten *clade are convergent and are derived from a free-living ancestor and a byssal attaching ancestor, respectively. Last, nestling of *Pedum spondyloideum *is a unique life habit in our phylogeny and originated from a byssal attaching ancestor. Ancestral state estimation is congruent when using the ML topology (Additional file 3, Figure S1), with one exception. The placement of *Nodipecten subnodosus *as the sister taxon to *E. chazaliei *in the ML topology creates a unique transition from the recessing condition to a free-living state not observed on the BI topology (data not shown).

Last, we examined whether transitions between life habit states were evolutionarily constrained. Without constraint, we would expect that each state would be equally likely to give rise to any of the other state. However, the byssal attaching gave rise to significantly more of these transitions, while the other states appear to be nearly fixed once they arise (*X*^2 ^= 37.003; d.f. = 5; p < 0.001). Even when we combined the nestling and cementing categories as "permanent attachers" to reduce the number of categories with a low number of observations, the byssal life habit is still significantly more likely to be the evolutionary progenitor of all other states (*X*^2 ^= 27.999; d.f. = 4; p < 0.001).

## Discussion

While patterns of convergence and parallelism are well-documented in vertebrate groups [3,10,14-16,18-20], less is known about such patterns in non-vertebrates. Our study represents a major contribution to understanding repeated patterns of evolution in a non-model invertebrate group, the Pectinidae. The complex evolutionary history of scallops involves multiple origins of life habit phenotypes, with five of the six life habits evolving at least twice during the diversification of the family. Byssal attachment was not only the most common life habit in scallops, but was the ancestral condition to significantly more of the habit transitions than any other category. Interestingly, gliding evolved independently at least four times through both convergent and parallel evolution implying that there is strong positive selection for this life habit. Thus the patterns revealed in this study, a limited number of possible evolutionary transitions, and the evolution of repeated phenotypes, correspond closely to what is expected for phenotypes under strong selection and functional constraint [54-56].

Byssal attachment and the subsequent loss of the byssal apparatus may have had a profound affect on the evolution and phenotypic diversification of the Pectinidae. All pectinid species have a byssate stage to secure the post-larval scallop to a substrate while metamorphosing into its adult form, and the majority of scallop species (5:1) retain this early ontogenetic condition into sexual maturity [49]. Our results indicate that byssal attaching is the most common life habit in extant scallop species and byssal attachers gave rise to significantly more life habit classes than any other state. Furthermore, we observed that particular transitions between states are unidirectional, while other transitions never occur. For example, cementing only occurs as a derived state. In contrast, the other life habit classes, byssal attaching, free-living, recessing, and to a lesser extent gliding, are both ancestral states and transitional outcomes. This bias in the types of observed life habit transitions may indicate a restriction in possible evolutionary outcomes for certain states due to the degree or complexity of physiological changes needed to transition from one life habit to another.

One possible constraint on the lability of a given life habit state may be the degree of morphological specialization of the shell. If shell morphology can restrict life habit transitions, we would expect the greatest number of transitions to occur between classes with most similar shell shapes (i.e. the smallest phenotypic distance). Qualitatively, byssal attachers and free-living species possess the most similar shell shapes, and we detected the greatest number of transitions (six) between these two classes. Additionally, both byssal attaching and free-living habits are epifaunal, allowing a simple transition from temporary attachment to non-attachment on a substrate - no specializations in habitat use required. Other life habit classes are associated with a dramatic change in shell morphology and/or specialized habitat use (e.g., from epifaunal to semi-epifaunal). For instance, distantly-related gliding species (*A*. *pleuronectes *and "*A*." *balloti*, Figure 1) share a similar lightweight, smooth, symmetrical shell. This convergent morphology [27] may restrict the ability of gliders to transition into a different state. Likewise, permanently attached species that cement to a substrate also may possess specific physiological traits that may prohibit life habit transitions.

Based on these observations, it would appear that some life habit classes are evolutionary dead ends. To examine this hypothesis it is important to consider whether all life habit classes have had sufficient time to serve as progenitor states. It may be that because lineages exhibiting byssal attaching are the most ancestral and widespread in the Pectinidae, sufficient time has passed to allow opportunities to generate other life habits, whereas "younger" lineages from the Miocene, such as those exhibiting gliding [29,57], may not have not had enough time to diversify. The cementing life habit seems to support of the hypothesis that some states are "dead ends." This state is old (Jurassic) and appears to have been more common during the Jurassic and Early Cretaceous periods than at present [58]. This suggests that although there may have been ample opportunities for the cementing life habit to function as a progenitor state, these lineages either were unable to transition to another life habit or went extinct before a transition.

Our ancestral state reconstruction analysis identified the minimum number of transitions on the tree, but due to incomplete taxonomic sampling, our analysis may not have detected all life habit transitions. However, the majority (58%) of the life habit transitions discussed in this study occur in clades that were most densely sampled (see dashed boxes in Figure 1). So far, the phylogenetic relationships within these clades generally follow the currently accepted taxonomic classification of scallops. In the remaining clades where taxonomic sampling is less complete, the majority of the unsampled taxa belong to the tribes Chlamydini (75 species) and Mimachlamydini (25 species). While the genera within these tribes largely are nonmonophyletic in our analyses, an increase in sampling may alter some phylogenetic relationships. However, since the majority of these taxa are byssate [29] it is unlikely that the addition of these species will alter the main conclusions of this work.

## Conclusions

Our study suggests that scallops have iteratively evolved similar life habit types. Previous authors have hypothesized that morphological evolution in the Pectinidae is highly repetitive, with particular shell forms representing putative adaptations to specific living habits [29,30,59]. Our results support this hypothesis, but the role of shell morphology needs to be further studied. Because life habit and shell morphology are closely linked [30,60], a formal test of the association between life habit and shell forms relative to pectinid ecology is needed. Recently, Serb *et al*. [27] identified substantial convergence of shell morphology in a subset of gliding scallop species which suggests that iterative morphological evolution may be more prevalent in the family than previously thought. Further investigations into the convergence of shell morphology and life habit could provide insight into what compensatory changes in morphology are required to allow transitions between life habits.

## Authors' contributions

AA, LP, and JMS conceived the study. AA (23%), LP (66%) and JMS (11%) collected data and all authors were involved in the analyses. AA, LP and JMS wrote the paper. All authors have read and approved the manuscript.

## Supplementary Material

Additional file 1 **Genbank accession numbers**. Genbank accession numbers, locality information, and specimen identification numbers given for all gene sequences in the analysis. Click here for file

Additional file 2 **Life habit assignment for material examined**. Life habit assignment for all taxa used in the analysis. Life habit data were assembled through a review of the literature and supplemented with the personal observations of collectors. Click here for file

Additional file 3 **Maximum likelihood phylogram of the Pectinidae**. Bootstrap support values (> 50%) above respective nodes. The Pectinidae is boxed in grey. Each hatch mark on outgroup branches indicates a reduction of branch length by 1 scale bar (0.3) length. Click here for file
