# Supplementary material for: Convergent and parallel evolution in life habit of the scallops (Bivalvia: Pectinidae)
Source: BMC Evol Biol. 2011 Jun 14;11:164. doi: 10.1186/1471-2148-11-164 (PMC3129317; doi:10.1186/1471-2148-11-164)
Supplement: Additional file 1 — Genbank accession numbers. Genbank accession numbers, locality information, and specimen identification numbers given for all gene sequences in the analysis. [file 1471-2148-11-164-S1.DOCX]

**Additional File 1: Table 1** Genbank accession numbers

**Genbank Accession Numbers of Gene Sequences**

**ID Number Locality 12S rRNA 16S rRNA Histone 3 28S rRNA**

amandi E1 Panama HM485575 HM485576 HM485577 HM485578

amandi E2 Panama HM535651 HM535652 HM535653 HM535654

AMNH 298075_1 unknown EU379406 EU379460 EU379514 HM630528

antillarum unknown HM535656 HM535657 HM535658 HM535659

asperrima 1 Hobart, Tasmania, Australia HM540080 HM540081 HM540082 HM540083

asperrima 3 Hobart, Tasmania, Australia HM540084 HM540085 HM540086 HM540087

balloti 1 Bundaberg, Queensland, Australia HM540088 HM540089 HM540090 HM540091

balloti 2 Bundaberg, Queensland, Australia EU379379 EU379433 EU379488 HM540092

balloti 3 Bundaberg, Queensland, Australia EU379380 EU379434 EU379489 HM540093

balloti 4 Bundaberg, Queensland, Australia HM540094 HM540095 HM540096 HM540097

balloti 5 Bundaberg, Queensland, Australia HM540098 HM540099 HM540100 HM540101

behringiana Alaska, USA FJ263632 FJ263641 FJ263661 FJ263650

bifrons 1 Tasmania, Australia HM561991 HM561992 HM561993 HM561994

bifrons 2 Tasmania, Australia HM561995 HM561996 HM561997 HM561998

caurinus Alaska, USA FJ263633 FJ26642 FJ263662 FJ263651

chazaliei Gulfo de Los Mosquitos, Panama EU379382 EU379436 EU379490 HM561999

colbecki* Terra Nova Bay, Antarctica EU379383 EU379437 EU379491 FJ263652

cruentus HPC Tateyama City, Chiba, Japan HM600761 HM600754 HM600734 HM600747

cuneata Tateyama City, Chiba, Japan HM622702 HM622703 HM622704 HM622705

farreri 1 Aquaculture Facility in Qindao, China HM622677 HM622678 HM622679 HM622680

farreri 2 Aquaculture Facility in Qindao, China HM622681 HM622682 HM622683 HM622684

fumatus 1 Hobart, Tasmania, Australia HM622689 HM622690 HM622691 HM622692

fumatus 2 Hobart, Tasmania, Australia HM622693 HM622694 HM622695 HM622696

gibbus 2 Harrington Sound, Bermuda EU379388 EU379442 EU379496 HM622697

gibbus 3 Harrington Sound, Bermuda EU379389 EU379443 EU379497 HM622698

gigantea Santa Barbara, California, USA FJ263635 FJ263644 FJ263664 FJ263654

hastata San Juan Island, Washington, USA FJ263639 FJ263648 FJ263667 FJ263658

HPC 556 Kyonan-cho, Chiba, Japan HM622710 HM622711 HM622712 HM622713

HPC 663 Hitachi City, Ibaraki, Japan HM630488 HM630489 HM630490 HM630491

HPC 735 Kasasa-cho, Kagoshima, Japan HM600762 HM600755 HM600735 HM600748

HPC 771 Bonotsu City (Minanisatsuma), Kagoshima, Japan HM630483 HM630484 HM630485 HM630486

HPC 1578 Miura City, Kanagawa, Japan HM622673 HM622674 HM622675 HM622676

irradians Gulf Marine Specimens Laboratory, USA EU379392 EU379446 EU379500 HM622700

islandica Quebec, Canada FJ263637 FJ263646 FJ263666 FJ263656

japonicum Oyano Island, Kumamoto, Japan HM622706 HM622707 HM622708 HM622709

Laevichlamys Japan HM630469 HM630470 HM630471 HM630472

latiauratus Goleta Pier, Santa Barbara, California, USA EU379393 EU379447 EU379501 HM622714

lemniscata 1 Tateyama City, Chiba, Japan HM622715 HM622716 HM622717 HM622718

lemniscata 2 Tateyama City, Chiba, Japan HM622719 HM622720 HM622721 HM622722

magellanicus Georges Bank, USA FJ263638 FJ263647 EU379506 FJ263657

maximus 1 Millport, Scotland EU379400 EU379454 EU379508 HM630545

multistriata 1 Gallicia, Spain EU379403 EU379457 EU379511 HM630539

multistriata 2 Gallicia, Spain HM630535 HM630536 HM630537 HM630538

nipponensis Kitaibaraki City, Ibaraki, Japan HM622685 HM622686 HM622687 HM622688

nobilis Kami-amakusa, Kumamoto, Japan HM630531 HM630532 HM630533 HM630534

novaezeland 2 Mercury Cove, Great Mercury Island, New Zealand EU379404 EU379458 EU379512 HM630530

novaezeland 3 Mercury Cove, Great Mercury Island, New Zealand EU379405 EU379459 EU379513 HM630529

opercularis 1 Millport, Scotland EU379408 EU379462 EU379516 HM630527

opercularis 2 Millport, Scotland EU379409 EU379463 EU379517 HM630526

ornata 1 Collao, Puerto Rico, USA HM630379 HM630380 HM630381 HM630382

ornata 2 Collao, Puerto Rico, USA HM630375 HM630376 HM630377 HM630378

papyraceum 1 Gulf of Mexico, USA HM630371 HM630372 HM630373 HM630374

patagonica J3 Chile EU379412 EU379466 EU379520 HM630524

patagonica J6 Chile HM630520 HM630521 HM630522 HM630523

perulus 1 Panama EU379413 EU379467 EU379521 HM630515

perulus 2 Panama EU379414 EU379468 EU379522 HM630514

pleuronectes 1 Rayong Province, Thailand EU379415 EU379469 EU379523 HM630508

pleuronectes 3 Rayong Province, Thailand HM630504 HM630505 HM630506 HM630507

pleuro QLD1 Queensland, Australia HM630500 HM630501 HM630502 HM630503

pleuro QLD2 Queensland, Australia HM630496 HM630497 HM630498 HM630499

plica Tateyama, Chiba, Japan HM630435 HM630436 HM630437 HM630438

purpuratus H3 Tongoy Bay, Chile EU379417 EU379471 EU379525 HM630495

pusio 1 Gallicia, Spain HM600764 HM600757 HM600737 HM600750

pusio 2 Gallicia, Spain HM600765 HM600758 HM600738 HM600751

rubida San Juan Island, Washington, USA FJ263636 FJ263645 FJ263665 FJ263655

senatoria 1 Gulf of Thailand, Thailand HM630479 HM630480 HM630481 HM630482

septem 2 Millport, Scotland EU379420 EU379474 EU379528 FJ263659

septem 3 Millport, Scotland EU379421 EU379475 EU379529 HM630477

squamata Tateyama City, Chiba, Japan HM630444 HM630445 HM630446 HM630447

subnodosus 9P Panama EU379427 EU379481 EU379535 HM630434

subnodosus 3M Baja California, Mexico HM630430 HM630431 HM630432 HM630433

UF280376 Sulawsi Island, Indonesia not seq'd HM630492 HM630493 HM630494

UF281663 Phuket, Thailand HM630391 HM630392 HM630393 HM630394

UF282407 Guam, USA EU379422 EU379476 EU379530 HM630456

UF282416 Guam, USA HM630461 HM630462 HM630463 HM630464

UF286387 Oman HM600763 HM600756 HM600736 HM600749

UF287521 Guam, USA EU379399 EU379453 EU379507 HM630546

UF288930 Guam, USA HM630510 HM630511 HM630512 HM630513

UF289624 Panama HM630541 HM630542 HM630543 HM630544

UF289879 Monroe County, Florida, USA EU379416 EU379470 EU379524 HM600740

UF292105 Viti Levu Island, Fiji EU379410 EU379464 EU379518 HM630525

UF292110 Viti Levu Island, Fiji HM630465 HM630466 HM630467 HM630468

UF292821 West Masirah, Oman HM630422 HM630423 HM630424 HM630425

UF295809 Saipan Island, Mariana Islands EU379401 EU379455 EU379509 HM630540

UF296052 Viti Levu Island, Fiji EU379396 EU379450 EU379504 HM630548

UF296350 Cocos-Keeling Island, Australia EU379384 EU379438 EU379492 HM600739

UF296996 Western Australia, Australia HM630439 HM630440 HM630441 HM630442

UF297000 Tanzania HM630473 HM630474 HM630475 HM630476

UF309990 Taiwan HM562000 HM562001 HM562002 HM562003

UF310406 Milne Bay, Papua New Guinea EU379429 EU379483 EU379537 HM622701

UF313444 Philippines HM630395 HM630396 HM630397 HM630398

UF313459 Philippines GU953232 GU953234 GU953233 HM630478

UF322180 Bismark Archipelago, Papua New Guinea EU379385 EU379439 EU379493 HM535655

UF322550 West of New Briton, Papua New Guinea EU379424 EU379478 EU379532 HM600743

UF323764 Florida Straits, Florida, USA EU379411 EU379465 EU379519 HM600741

UF323809 Bismark Archipelago, Papua New Guinea EU379397 EU379451 EU379505 HM630547

UF329089 Port Elizabeth, South Africa HM630426 HM630427 HM630428 HM630429

UF332786 North Cape, New Zealand HM600760 HM600753 HM600733 HM600746

UF343587 Stingray Shoals, Mariana Islands HM630452 HM630453 HM630454 HM630455

UF348872 Sullivan's Patches, Papua New Guinea HM630448 HM630449 HM630450 HM630451

UF351155 Florida, USA EU379391 EU379445 EU379499 HM622699

UF351301 Florida, USA EU379419 EU379473 EU379527 HM630487

UF351954 Okinawa, Japan EU379426 EU379480 EU379534 HM630443

UF352373 Ie Island, Okinawa, Japan EU379387 EU379441 EU379495 HM622672

UF352374 Okinawa, Japan HM630457 HM630458 HM630459 HM630460

UF352388 Okinawa, Japan HM630553 HM630554 HM630555 HM630556

UF367478 Florida Keys, Florida, USA EU379386 EU379440 EU379494 HM600745

UF367487 Florida Keys, Florida, USA EU379423 EU379477 EU379531 HM600742

UF367882 Muscat, Qurm, Oman HM630549 HM630550 HM630551 HM630552

UF368676 Shefa Province, Vanuatu EU379425 EU379479 EU379533 HM600744

UF369432 Fiji HM540103 HM540104 HM540105 HM540106

UF371263 Gulf of Panama, Panama HM630516 HM630517 HM630518 HM630519

UF371875 East of Naos, Panama EU379381 EU379435 EU379487 HM540102

vancouv* USA HM630418 HM630420 HM630416 HM630417

varia varia 1 Gallicia, Spain EU379428 EU379482 EU379536 HM630415

varia varia 2 Gallicia, Spain HM630411 HM630412 HM630413 HM630414

ventricosus 1 Bahia Magdalena, Baja California Sur, Mexico HM630407 HM630408 HM630409 HM630410

vesiculosus 1 Miura City, Kanagawa, Japan HM630403 HM630404 HM630405 HM630406

vesiculosus 2 Miura City, Kanagawa, Japan HM630399 HM630400 HM630401 HM630402

vogdesi 1 Bahia Magdalena, Baja California Sur, Mexico HM630387 HM630388 HM630389 HM630390

yessoensis 1 Mutsu Bay, Aomori, Japan FJ263640 FJ263649 FJ263668 FJ263660

yessoensis 2 Mutsu Bay, Aomori Japan HM630383 HM630384 HM630385 HM630386

ziczac 1 Harrington Sound, Bermuda EU379430 EU379484 EU379538 HM630509
