# Supplementary material for: Convergent and parallel evolution in life habit of the scallops (Bivalvia: Pectinidae)
Source: BMC Evol Biol. 2011 Jun 14;11:164. doi: 10.1186/1471-2148-11-164 (PMC3129317; doi:10.1186/1471-2148-11-164)
Supplement: Additional file 2 — Life habit assignment for material examined. Life habit assignment for all taxa used in the analysis. Life habit data were assembled through a review of the literature and supplemented with the personal observations of collectors. [file 1471-2148-11-164-S2.DOCX]

**Additional File2: Table 2.** Life habit assignment

**Species Life Habit ID Number***

**Family Pectinidae**

**Subfamily** Camptopectinae

*Delectopecten randolphi* Dall, 1897 free-living (T. Haga , Trawled, fine sand and mud bottom ) HPC 663

*Delectopecten vancouverensis* (Whiteaves, 1893) byssal attach [1] vancouv

**Subfamily** Chlamydinae

**Tribe** Adamussiini

*Adamussium colbecki* (Smith, 1902) glide [2, 3] colbecki

**Tribe** Chlamydini

*Azumapecten farreri farreri* (Jones & Preston, 1904) byssal attach [4, 5] “Zhikong Scallop” farreri 1

farreri 2

*Azmapecten farreri nipponensis* (Kuroda, 1932) byssal attach (T. Haga, attached to oyster shell, lower intertidal zone) nipponensis

*Chlamys behringiana* (Middendorff, 1849) byssal attach (inferred from shell morphology) behringiana

*Chlamys hastata* (Sowerby II, 1842) byssal attach [4] hastata

*Chlamys islandica* (Müller, 1776) byssal attach [3, 4, 6] islandica

*Chlamys rubida* (Hinds, 1845) byssal attach [4] rubida

*Coralichlamys madreporarum* (Sowerby II, 1842) byssal attach [7] UF296052

UF323809

*Equichlamys* *bifrons* (Lamarck, 1819) free-living [4, 8] bifrons 1

bifrons 2

*Laevichlamys cuneata* (Reeve, 1853) byssal attach [9], (T. Haga, gravel bottom attached, gillnet) UF310406

cuneata

*Laevichlamys lemniscata* (Reeve, 1853) byssal attach (T. Haga, gravel bottom attached, gillnet) lemniscata 1

lemniscata 2

*Laevichlamys* sp. byssal attach (T. Haga, gravel bottom attached, gillnet) Laevichlamys

*Laevichlamys squamosa* (Gmelin, 1791) byssal attach [9] UF351954

*Pascahinnites coruscans coruscans* (Hinds, 1845) byssal attach [9] UF296350

*Pedum spondyloideum* (Gmelin, 1791) nestle [7] UF343587

UF348872

*Scaeochlamys livida* (Lamark, 1819) byssal attach [10] UF367882

*Scaeochlamys squamata* (Gmelin, 1791) byssal attach (T. Haga, gravel bottom attached, gillnet) squamata

*Semipallium dianae* (Crandall, 1979) byssal attach [10] UF352388

*Semipallium* *dringi* (Reeve, 1853) byssal attach [11] UF352373

*Semipallium marybellae* Raines, 1996 byssal attach (inferred from shell morphology) UF287521

*Semipallium schmeltzii* Dunker *in* Küster & byssal attach (T. Haga & Y. Kano, attached to stone underside) HPC 771

Kobelt, 1888

*Talochlamys multistriata* (Poli, 1795) byssal attach [12] multistriata 1

multistriata 2

*Talochlamys pusio* (Linnaeus, 1758) cement [3, 12] pusio 1

pusio 2

*Talochlamys tinctus* (Reeve, 1853) byssal attach (inferred from shell morphology) UF329089

*Veprichlamys empressae* Kuroda, Habe & byssal attach (T. Haga, attached to a stone, dredged) HPC 1578

Oyama, 1971

*Veprichlamys jousseaumei* (Bavayi, 1904) byssal attach (T. Haga, attached to sunken wood on mud bottom) HPC 556

*Zygochlamys* *amandi* (Hertlein, 1935) byssal attach (inferred from shell morphology) amandi E1

amandi E2

*Zygochlamys* *patagonica* (King & Broderip, 1832) byssal attach [4] patagonica J3

patagonica J6

**Tribe** Crassadomini

*Caribachlamys mildredae* (Bayer, 1941) byssal attach [12] UF289624

*Caribachlamys ornata* (Lamarck, 1819) byssal attach [12] ornata 1

ornata 2

*Caribachlamys sentis* (Reeve, 1853) byssal attach [12] UF313459

*Crassadoma* *gigantea* (Gray, 1825) cement [4, 13] gigantea

**Tribe** Fortipectinini

*Mizuhopecten* *yessoensis* (Jay, 1857) recess [2-4] yessoensis 1

yessoensis 2

*Patinopecten caurinus* (Gould, 1850) recess [4, 14] caurinus

**Tribe** Mimachlamydini

*Mimachlamys* *asperrima* (Lamarck, 1819) byssal attach [3, 4, 8] asperrima 1

asperrima 3

*Mimachlamys* *cloacata* (Reeve, 1853) free-living [10] UF309990

*Mimachlamys nobilis* Reeve, 1852 byssal attach [4], (T. Haga, lower intertidal zone, attached to pebble) nobilis

*Mimachlamys senatoria* Gmelin, 1791 byssal attach [4, 7] senatoria 1

*Mimachlamys* sp. byssal attach (inferred from shell morphology) UF297000

*Mimachlamys townsendi* (Sowerby III, 1895) byssal attach [15] UF292821

*Mimachlamys* varia *varia* (Linnaeus, 1758) byssal attach [3, 4] varia varia 1

varia varia 2

*Spathochlamys* *benedicti* (Verrill & Bush in byssal attach [12] UF369432

Verrill, 1897)

**Tribe** Palliolini

*Placopecten magellanicus* (Gmelin, 1791) glide [3] magellanicus

*Pseudamussium septemradiatus* Müller, 1776 free-living [16] septem 2

septem 3

**Subfamily** Pectininae

**Tribe** Decatopectinini

*Anguipecten picturatus* Dijkstra, 1995 free-living (inferred from shell morphology) UF288930

*Bractechlamys* *antillarum* (Récluz, 1853) byssal attach [17] antillarum

*Bractechlamys* *vexillum* (Reeve, 1853) free-living [11] UF313444

UF281663

*Decatopecten plica* (Linnaeus, 1758) free-living [7] plica

*Decatopecten radula radula* (Linnaeus, 1758) free-living [10] UF280376

*Decatopecten* *strangei* (Reeve, 1852) free-living (inferred from shell morphology) UF296996

*Excellichlamys* *spectabilis* (Reeve, 1853) byssal attach [7, 9] UF282416

UF352374

*Gloripallium* *pallium* (Linnaeus, 1758) byssal attach [7] UF292105

*Gloripallium* *speciosum* (Reeve, 1853) byssal attach [10] UF292110

*Mirapecten* *mirificus* (Reeve, 1853) free-living [11] UF295809

*Mirapecten rastellum* (Lamarck, 1819) byssal attach [7] UF282407

*Nodipecten* *subnodosus* (Sowerby I, 1835) free-living (Dr. Ana M. Ibarra, personal communication) subnodosus 9P

subnodosus 3M

**Tribe** Pectinini

*Aequipecten* *glyptus* (Verrill, 1882) free-living (inferred from shell morphology) UF351155

*Aequipecten* *opercularis* (Linnaeus, 1758) free-living [3, 4] opercularis 1

opercularis 2

"*Amusium*" *balloti* (Bernardi, 1861) glide [3, 18] balloti 1

balloti 2

balloti 3

balloti 4

balloti 5

"*Amusium*" *japonicum* *japonicum* (Gmelin, 1791) glide [4], (N. Deguchi, sandy bottom, gillnet) japonicum

"*Amusium*" *papyraceum* glide [4] papyraceum 1

*Amusium* *pleuronectes* (Linnaeus, 1758) glide [3, 4, 19] pleuronectes 1

pleuronectes 3

pleuro QLD1

pleuro QLD2

*Argopecten* *gibbus* (Linnaeus, 1758) free-living [3, 4] gibbus 2

gibbus 3

*Argopecten irradians* *irradians* (Lamarck, 1819) free-living [3, 4, 20] irradians

*Argopecten* *nucleus* (Born, 1778) free-living [4] AMNH

298075_1

*Argopecten* *purpuratus* (Lamarck, 1819) free-living [4, 21] purpuratus H3

*Argopecten* *ventricosus* (Sowerby II, 1842) free-living [4] ventricosus 1

*Cryptopecten vesiculosus* (Dunker, 1877) free-living [15], (C. Kobayashi, gravel bottom, dredged) vesiculosus 1

vesiculosus 2

*Euvola* *chazaliei* (Dautzenberg, 1900) recess (inferred from shell morphology) chazaliei

*Euvola* *perula* (Olsson, 1961) recess (inferred from shell morphology) UF371263

perula 1

perula 2

*Euvola raveneli* (Dall, 1898) recess (inferred from shell morphology) UF351301

*Euvola vogdesi* (Arnold, 1906) recess [4] vogdesi 1

*Euvola* *ziczac* (Linnaeus, 1758) recess [4, 22] ziczac 1

*Leptopecten* *bavayi* (Dautzenberg, 1900) byssal attach [17] UF371875

*Leptopecten* *latiauratus* (Conrad, 1837) byssal attach [23] latiauratus

*Pecten fumatus* Reeve, 1852 recess [3, 4] fumatus 1

fumatus 2

*Pecten* *maximus* (Linnaeus, 1758) recess [3, 4] maximus 1

*Pecten* *novaezelandiae* Reeve, 1852 recess [3, 4] novaezeland 2

novaezeland 3

**OUTGROUPS**

**Family Limidae**

*Ctenoides* *annulatus* (Lamarck, 1819) byssal attach [24] UF322180

*Ctenoides* *mitis* (Lamarck, 1807) byssal attach [24] UF367478

*Lima colorata zealandica* Sowerby, 1876 byssal attach [24] UF332786

*Lima sowerbyi* Deshayes, 1863 byssal attach [24] UF286387

**Family Propeamussidae**

*Parvamussium* *pourtalesianum* (Dall, 1886) byssal attach [15] UF323764

*Propeamussium* *dalli* (Smith, 1885) byssal attach [15] UF289879

*Propeamussium sibogai* (Dautzenberg & Bavay, 1904) byssal attach [15], (T. Haga, sandy-muddy bottom, shrimp trawl) HPC 735

**Family Spondylidae**

*Spondylus cruentus* Lischke, 1868 cement (T. Haga, gravel bottom, sessile on a mud rock, gillnet) cruentus HPC

*Spondylus* *ictericus* Reeve, 1856 cement [25] UF367487

*Spondylus nicobaricus* Schreibers, 1793 cement [26] UF322550

*Spondylus squamosus* Schreibers, 1793 cement [26] UF368676

*UF, Florida Museum of Natural History (University of Florida, Gainesville); HPC, field collection number for Takuma Haga.

1. Whiteaves JF: **Notes on some marine Invertebrata from th coast of British Columbia.** *Ottawa Naturalist* 1893, **9**(7):133-137.

2. Sakurai I, Seto M: **Movement and orientation of the Japanese scallop *Patinopecten yessoensis* (Jay) in response to water flow.** *Aquaculture* 2000, **181**:269-279.

3. Brand AR: **Scallop Ecology: Distributions and Behavior. In: S. E. Shumway & J. Parsons (Eds.), Scallops: Biology, Ecology and Aquaculture. Elsevier Science, 2nd edition.** 2006, **35**:651-713.

4. Minchin D: **Introductions: some biological and ecological characteristics of scallops.** *Aquatic Living Resources* 2003, **16**:521-532.

5. Guo X, Luo Y: **Scallop Culture in China. In: S. E. Shumway & J. Parsons (Eds.), Scallops: Biology, Ecology and Aquaculture. Elsevier Science, 2nd edition.** 2006, **35**:1143-1161.

6. Gilkinson KD, Gagnon JM: **Substratum associations of natural populations of Iceland scallops, *Chlamys islandica* Müller 1776, on the northeastern Grand Bank of Newfoundland.** *American Malacological Bulletin* 1991, **9**:59-67.

7. Dijkstra HH, Knudsen J: **Some Pectinoidea (Mollusca: Bivalvia: Propeamussiideae, Pectinidae) of the Red Sea.** *Molluscan Research* 1998, **19**(2):43-104.

8. Styan C, Butler AJ: **Asynchronus patters of reproduction for the sympatric scallops *Chlamys bifrons* and *Chlamys asperrima* (Bivalvia: Pectinidae) in South Australia.** *Marine and Freshwater Research* 2003, **54**(1):77-86.

9. Dijkstra HH, Moolenbeek RG: **Some Pectinoidea (Bivalvia: Propeamussiidae and Pectinidae) from the Berau Islands (East Kalimantan, Indonesia).** *Venus* 2008, **67**(1-2):15-26.

10. Dijkstra HH: **A contribution to the knowledge of the pectinacean Mollusca (Bivalvia: Propeamussiidae, Entoliidae, Pectinidae) from the Indonesian Archipelago.** *Zoologische Verhandelingen, Leiden* 1991, **271**:1-57.

11. Dijkstra HH: **Pectinoidea (Mollusca: Bivalvia: Pectinidae: Propeamussiidae) from Hansa Bay, Papua New Guinea.** *Molluscan Research* 1998, **19**(1):11-52.

12. Waller TR: **The evolution of "*Chlamys*" (Mollusca: Bivalvia: Pectinidae) in the tropical western Atlantic and eastern Pacific.** *American Malacological Bulletin* 1993, **10**(2):195-249.

13. Lauzier RB, Bourne NF: **Scallops of the west coast of North America. In: S. E. Shumway & J. Parsons (Eds.), Scallops: Biology, Ecology and Aquaculture. Elsevier Science, 2nd edition.** 2006, **35**:965-989.

14. Masuda MM, Stone RP: **Biological and spatial characteristics of the weathervane scallop *Patinopecten caurinus* at Chiniak Gully in the Central Gulf of Alaska.** *Alaska Fishery Research Bulletin* 2003, **10**(2):104-118.

15. Waller TR: **The ctenolium of scallop shells: functional morphology and evolution of a key family-level character in the Pectinacea (Mollusca: Bivalvia)**. *Malacologia* 1984, **25**(1):203-219.

16. Allen JA: **Observations on the epifauna of the deep-water muds of the Clyde Sea area, with special reference to Chlamys septemradiata (Müller).** *Journal of Animal Ecology* 1953, **22**:240-260.

17. Smith JT, Jackson BC: **Ecology of extreme faunal turnover of tropical American scallops**. *Palleobiology* 2009, **35**(1):77-93.

18. Joll LM: **Swimming behaviour of the saucer scallop *Amusium balloti* (Mollusca: Pectinidae)**. *Marine Biology* 1989, **102**:299-305.

19. Morton B: **Swimming in Amusium pleuronectes (Bivalvia: Pectinidae)**. *J Zool Lond* 1980, **190**:375-404.

20. Pohle D, Bricelj V, Garcia-Esquivel Z: **The eelgrass canopy: an above-bottom refuge from benthic predators for juvenile bay scallops *Argopecten irradians*.** *Marine Ecology Progress Series* 1991, **74**:47-59.

21. Piquimil RN, Figueroa LS, Contreras OC, Avendaño M: **Fisheries and aquaculture: Chile. In: Scallops: Biology, Ecology and Aquaculture S.E. Shumway, Editor, Developments in Aquaculture and Fisheries Science Vol. 21, Elsevier, New York, pp. 1001–1015.** 1991.

22. Vélez A, Freites L, Himmelman JH, Senior W, Marin N: **Growth of the tropical scallop, *Euvola* (*Pecten*) *ziczac*, in bottom and suspended culture in the Golfo de Cariaco, Venezuela.** *Aquaculture* 1995, **136**(3-4):257-276.

23. Morton B: **The biology and functional morphology of *Leptopecten latiauratus* (Conrad, 1837): An "opportunistic" scallop.** *Veliger* 1994, **37**(1):5-22.

24. Mikkelsen PM, Bieler R: **Systematic revision of the western Atlantic file clams, *Lima* and *Ctenoides* (Bivalvia: Limoida: Limidae)**. *Invertebrate Systematics* 2003, **17**:667-710.

25. Perry LM, Schwengel JS: **Marine shells of the western coast of Florida. Ithaca, New York: Paleontological Research Institution. 318 pgs.** 1955.

26. Slack-Smith SM: **Order Ostreoida. In: Bessley, P. L., Ross, G. J. B., Wells, A. (Eds.), Mollusca: Southern Synthesis. Fauna of Australia, CSIRO Publishing, Melbourne, pp. 268-282.** 1998.
