# Supplementary figures and images for: Convergent and parallel evolution in life habit of the scallops (Bivalvia: Pectinidae)
Source: BMC Evol Biol. 2011 Jun 14;11:164. doi: 10.1186/1471-2148-11-164 (PMC3129317; doi:10.1186/1471-2148-11-164)

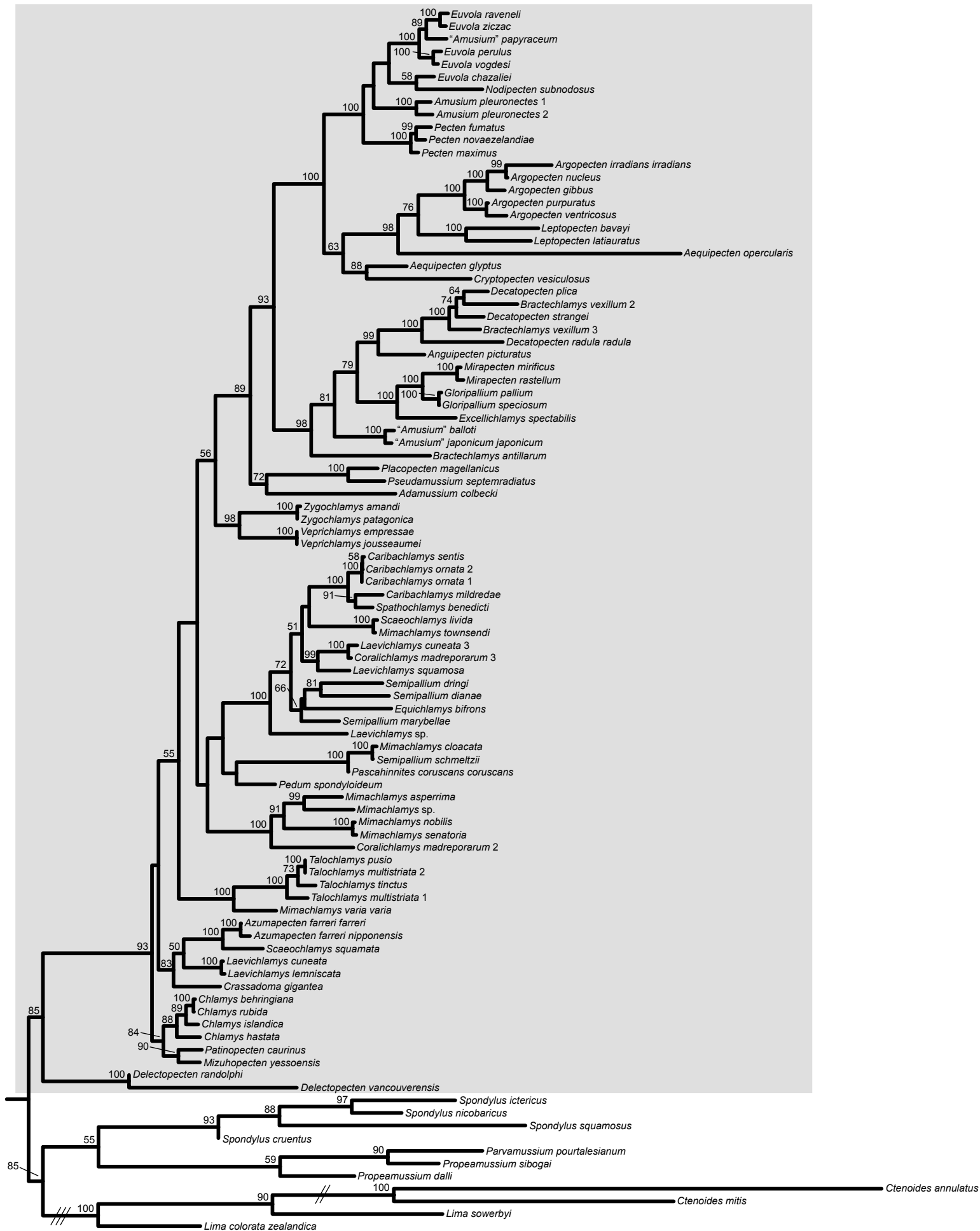

Supplement: Additional file 3 — Maximum likelihood phylogram of the Pectinidae. Bootstrap support values (> 50%) above respective nodes. The Pectinidae is boxed in grey. Each hatch mark on outgroup branches indicates a reduction of branch length by 1 scale bar (0.3) length. [file 1471-2148-11-164-S3.PDF]
